# Quality of Life, Its Determinants, and Psychiatric Comorbidities in Juvenile Myoclonic Epilepsy: A Cross-Sectional Observational Study From North India

**DOI:** 10.7759/cureus.69228

**Published:** 2024-09-11

**Authors:** Cankatika Choudhury, Akhil Sahib, Neera Chaudhry, Manushree Gupta, Shishir K Chandan, Sanghamitra Laskar

**Affiliations:** 1 Neurology, Govind Ballabh Pant Institute of Postgraduate Medical Education and Research, New Delhi, IND; 2 Psychiatry, Vardhman Mahavir Medical College and Safdarjung Hospital, New Delhi, IND; 3 Neurology, Vardhman Mahavir Medical College and Safdarjung Hospital, New Delhi, IND

**Keywords:** behavioural neurology, comprehensive epilepsy care, juvenile myoclonic epilepsy, psychiatric co-morbidity, quality of life (qol)

## Abstract

Background: Juvenile myoclonic epilepsy (JME) is a well-controlled genetic generalized epilepsy (GGE) syndrome with a favourable prognosis but the long-term outcome is still controversial due to the presence of personality traits, executive dysfunction, and psychiatric disorders inherent to this condition. Also, the existing literature on quality of life (QoL) in adolescent patients of JME is sparse. This study was done to assess the QoL, its determinants, and the presence of psychiatric comorbidities in JME.

Materials and methods: The study was a hospital-based observational cross-sectional study of 50 participants done over 18 months. Patients of JME aged over 11 years fulfilling the diagnostic and electroencephalographic criteria were included in the study. Adolescent and adult JME participants were interviewed with the Quality of Life in Epilepsy-Adolescents-48 (QOLIE-AD-48) and patient-weighted Quality Of Life in Epilepsy-31 (QOLIE-31-P), respectively, for assessment of QoL, the domains affected, and its impact on overall QoL. They were also screened for psychiatric disorders with Mini International Neuropsychiatric Interview 7.0.2 (M.I.N.I. 7.0.2), a brief diagnostic-structured interview that has modules for each diagnostic category. The Diagnostic and Statistical Manual of Mental Disorders, Fifth Edition (DSM-5) was used for further diagnostic categorization.

Results: Fifty patients with JME were enrolled. The mean age was 24.14 ± 7.7 years, of which 32 (64%) were female patients. The overall QOLIE-31-P score in adult JME participants was fair (62.29 ± 25.02). The impacted subdomains in adults were of seizure worry (47.73 ± 24.62) and cognitive functioning (46.41 ± 25.32). The mean QOLIE-48-AD score of adolescent JME study participants was fair (69.71 ± 13.13). The physical functioning (57.36 ± 18.94) and health perception (56.5 ± 16.9) domains were found to be impacted in adolescents. Five (10%) of the patients had anxiety and three (6%) of the participants had depression. A significant association was seen between the occurrence of generalized tonic-clonic seizure (GTCS) frequency (per year) and the presence of psychiatric comorbidity (p-value < 0.05).

Conclusion: JME may have a negative impact on overall QoL in both adolescents and adults despite adequate seizure control. Fear of seizure recurrence, cognitive issues, negative perception of health, and comorbid psychiatric disorders need to be addressed simultaneously and treated holistically as part of comprehensive epilepsy care to improve long-term outcomes.

## Introduction

Epilepsy is the oldest known neurological disorder of mankind and it is faced with stigmatization even in the modern-day world. It was believed to be caused by the wrath of the God of the Moon until its recognition as a primary brain disorder by Hippocrates [1]. Until the mid-20th century, there were discriminatory marriage laws, reduced access to health insurance, and job restrictions for people with epilepsy (PWE), leading to their social isolation. Addressing the quality of life (QoL) of PWE is now being recognized as an important component of comprehensive epilepsy care [1,2]. Juvenile myoclonic epilepsy (JME) or Janz syndrome is the most frequent idiopathic genetic generalized epilepsy (GGE) syndrome, accounting for about 3-11% of all adult and adolescent epilepsy cases [3]. Recent insights have shown that JME is in fact a complex disorder with changes in frontal circuits, the thalamus, and GABAergic transmission. It is not as well-controlled with valproate as it was thought to be around 67 years back. In fact, one-third of JME patients might have pseudo-drug resistance attributable to psychiatric comorbidities or other psychological factors [3]. Further, 74% of JME patients have been shown to have at least one major feature of unfavourable social outcomes [4]. In its first description, JME was described as “impulsive petit mal.” This may be further substantiated by a typical personality of indiscipline, impulsivity, and apathy [4]. Because of their inattentiveness, it is often challenging for these patients to follow an organized daily routine. Psychiatric associations, such as anxiety, mood disorders, and mild personality dysfunction, have been reported [3,5]. Also, drugs such as levetiracetam and topiramate may worsen coexisting psychiatric disturbances, and lamotrigine may worsen myoclonic jerks. These factors taken together may cause problems with drug compliance, seizure control, and societal integration. Most of the studies on epilepsy and its impact on QoL have focused on intractable epilepsy, particularly on temporal lobe epilepsy. Liaison neuropsychiatry is essential in the psychiatric care of neurological patients depending upon the complexity of cases to prevent injudicious use of health services and to curb medical expenses. To date, there is limited research on QoL, its determinants, and psychiatric associations in both adolescents and adults. We aimed to study QoL and psychiatric comorbidities in patients of JME. The primary objectives were to assess QoL in terms of psychological, social, and vocational aspects of life and to identify psychiatric disorders, if any.

## Materials and methods

Study setting

A hospital-based observational cross-sectional study was carried out from January 2019 to June 2020 in the Department of Neurology in collaboration with the Department of Psychiatry at Vardhman Mahavir Medical College and Safdarjung Hospital, New Delhi, India. Patients presenting to the neurology outpatient services and epilepsy clinic were screened. Fifty participants were enrolled. The sample size was calculated based on the study of Holtkamp et al. in 2014, where the mean value of patient-weighted Quality Of Life in Epilepsy-31 (QOLIE-31-P) in JME participants was 74.4 ± 2.6 [5]. Taking this value as a reference, the minimum required sample size with an estimate of QOLIE -31-P to be within 0.744 scores by taking the margin of error as 1% and 5% level of significance are 47. To reduce the margin of error, the total sample size taken was 50. Ethical clearance for this study was obtained from the Institutional Ethical Clearance Committee (vide IEC/VMMC-SJH/Thesis/December/12/2018). All study participants were explained about the purpose of the study. Confidentiality was assured to them, along with informed written consent for adults and assent from guardians for minors. The study complied with the Declaration of Helsinki.

Participants over 11 years who fulfilled the Class II diagnostic consensus criteria of JME were included in the study [6]. The criteria used were presence of myoclonic jerks predominantly on awakening, myoclonic jerks facilitated by sleep deprivation and stress or provoked by visual stimuli and praxis, and generalized tonic-clonic seizure (GTCS) preceded by myoclonic jerks along with EEG with normal background and at least one interictal generalized polyspike and wave discharge (PSWD) with or without myoclonic jerks, absence of mental retardation or deterioration, and age at onset between six and 25 years. Those with intellectual disability, history of meningitis/encephalitis, long-standing medical disorders, recent history (less than six months) of traumatic brain injury, and history of substance abuse were excluded.

Assessment

History was taken regarding sociodemographic details, age of onset, seizure type, frequency, control, and anti-seizure medications. Clinical examination was performed and recorded in a predesigned proforma. For the purpose of this study, control of epilepsy was defined as no occurrence of either myoclonic jerks or GTCS in the preceding six months from enrolment. The patients underwent 40 minutes of awake and sleep EEG recording on a 24-channel EEG machine (RMS) to look for generalized 4-6 Hz spike-wave and polyspike-wave discharges. A 3T MRI brain epilepsy protocol was also done to rule out any intracranial lesion.

Tools

Validated and permitted English and Hindi versions of the Quality of Life in Epilepsy-Adolescents-48 (QOLIE-AD-48) and the QOLIE-31-P questionnaires were used for adolescent and adult populations, respectively, for the assessment of QoL [7,8]. Adolescent JME participants aged ≤17 years were tested using the QOLIE-AD-48 [7]. Eight subdomains of QoL having 48 items were tested. These were epilepsy impact, memory, concentration, attitudes toward epilepsy, physical functioning, stigma, social support, school behaviour, health perceptions, and a total summary score. The higher the score, the better the QoL. Those aged over 18 years were evaluated with the patient-weighted QOLIE‐31-P version 2.0. Thirty items were tested in the following seven subscale domains: seizure worry, emotional well‐being, energy/fatigue, cognitive functioning, medication effects, overall QoL, and social functioning. Each domain was scored after calculating the mean score of responses within that domain. The overall score was the weighted average of all the domains’ scores, which were then converted to 0-100 according to a scoring manual [8]. QoL was categorized as follows: a poor score is 0-49, a fair score is 50-74, and a good score is 75-100.

They were screened for psychiatric disorders using the licensed and permitted Mini International Neuropsychiatric Interview (M.I.N.I. 7.0.2), a concise and structured psychiatric interview for all the major psychiatric disorders enlisted in the Diagnostic and Statistical Manual of Mental Disorders, Fifth Edition (DSM-5) and the International Classification of Diseases, 10th Revision (ICD-10) [9]. It has 16 modules, A to P, each corresponding to a diagnostic category. The categories include major depressive episodes, suicidality, manic and hypomanic episodes, panic disorder, agoraphobia, obsessive-compulsive disorder, posttraumatic stress disorder, alcohol use disorder, substance use disorder (non-alcohol), psychotic disorders and mood disorders with psychotic features, anorexia nervosa, bulimia nervosa, generalized anxiety disorder, anti-social personality disorder. All questions were rated and marked as yes or no. Those who were screened positive for any psychiatric disorder were further assessed with DSM-5 for current and lifetime diagnosis [10].

Statistical analyses

Data was entered into an MS Excel spreadsheet (Microsoft® Corp., Redmond, WA, USA) and analyzed using Statistical Package for Social Sciences (SPSS) version 21.0 (IBM Corp., Armonk, NY, USA). Categorical variables were expressed in number and percentage (%) and continuous variables as mean ± standard deviation (SD) and median. Quantitative variables were compared using independent t-tests between two groups and with analysis of variance (ANOVA) tests between three groups. Qualitative variables were compared using chi-square tests and Fisher’s exact tests. A p-value of <0.05 was considered statistically significant.

## Results

Participant characteristics

Fifty participants were enrolled in the study. They were between ages 15-52 years and the mean age was 24.14 ± 7.7 years; 26 (52%) belonged to the age group of 21-30 years, followed by 17 (34%) in the ≤ 20 years age group; 32 (64%) were females, and the male-female ratio was 1:1.8; 37 members (74%) of the study population belonged to the middle socioeconomic status according to the Kuppuswamy scale, and 10 (20%) belonged to the lower socioeconomic status; 15 members (30%) of the study population were married, nine (18%) were housewives, 21 (42%) were students, and four (8%) were school dropouts; 14 (28%) were employed and six (12%) were unemployed. For 48 (96%) of the participants, the age of onset (in years) of seizures was between 11 and 20 years, followed by an onset over 20 years in two (4%) of the participants. The mean age (± SD) of onset of seizures was 15.28 ± 2.9 years. The mean duration of epilepsy of the study subjects was 8.95 ± 6.88 years, with a duration between six and 10 years in 20 (40%) and a duration over 10 years in 15 (30%); 48 (96%) had both GTCS and myoclonic jerks, and all three seizure types, including absences, were seen in only one (2%). The GTCS frequency/year was less than one in 26 (52%), 1-3 in 19 (38%), and over 3 in five (10%). About 30 (60%) had uncontrolled epilepsy. EEG abnormalities included 4-6 Hz PSWDs with fronto-central predominance in 17 (34%) and 3-4 Hz spikes and wave discharges (SWD) and PSWD in 15 (30%). Epilepsy was not controlled in 30 (60%). The sociodemographic data and the clinical features are presented in Table 1.

**Table 1:** Sociodemographic and clinical characteristics of JME study participants Control of epilepsy has been defined as the absence of either myoclonic jerks or GTCS in the preceding six months. JME: juvenile myoclonic epilepsy; GTCS: generalized tonic-clonic seizure

Variables	Frequency (n = 50)	Percentage (%)
Age (years)		
≤20	17	34.00
21-30	26	52.00
>30	7	14.00
Gender		
Female	32	64.00
Male	18	36.00
Educational status		
School dropout	4	8.00
High school	21	42.00
College	8	16.00
Graduate/postgraduate	17	34.00
Socioeconomic status		
Low class	10	20.00
Middle class	37	74.00
Upper class	3	6.00
Religion		
Hindu	37	74.00
Jain	2	4.00
Muslim	7	14.00
Sikh	4	8.00
Occupation		
Employed	14	28.00
Housewife	9	18.00
Student	21	42.00
Unemployed	6	12.00
Marital status		
Married	15	30.00
Unmarried	35	70.00
Age of onset of seizures (years)		
11-20	48	96.00
>20	2	4.00
Duration of epilepsy (years)		
<5	15	30.00
6-10	20	40.00
11-15	9	18.00
>15	6	12.00
Seizure types		
GTCS, myoclonic jerks, absence seizure	1	2.00
GTCS	48	96.00
Myoclonic jerks	1	2.00
Seizure frequency (GTCS/year)		
<1 per year	26	52.00
1-3 per year	19	38.00
>3 per year	5	10.00
Control of epilepsy		
Yes	20	40.00
No	30	60.00

Thirty-six participants (72%) had good drug compliance and 29 (58%) were on polytherapy; 19 (38%) participants were on levetiracetam monotherapy, followed by valproate monotherapy in eight (16%), and 23 (46%) were on combination therapy; five (10%) reported irritability (Table 2).

**Table 2:** Distribution of anti-seizure medications used in JME study participants JME: juvenile myoclonic epilepsy

Drugs	Frequency (n = 50)	Percentage (%)
Clobazam	1	2.00
Levetiracetam	19	38.00
Levetiracetam and clobazam	4	8.00
Levetiracetam and clonazepam	1	2.00
Levetiracetam, clobazam, and clonazepam	1	2.00
Phenytoin	1	2.00
Phenytoin and levetiracetam	2	4.00
Phenytoin, phenobarbitone, and levetiracetam	1	2.00
Valproate	8	16.00
Valproate and clobazam	4	8.00
Valproate and levetiracetam	1	2.00
Valproate and levetiracetam	3	6.00
Valproate, clobazam, and lamotrigine	1	2.00
Valproate, levetiracetam, and clobazam	2	4.00
Valproate, phenytoin, and clobazam	1	2.00

QoL and its determinants

QOLIE-31-P

The mean (± SD) QOLIE-31-P score was fair (62.29 ± 25.02). The QOLIE-31-P score was fair in 16 (37.21%), good in 15 (34.88%), and poor in 12 (27.91%) of the 43 adult JME participants. In subdomain analysis, the energy perception (65.56 ± 16.85), overall well-being (63 ± 24.11), social functioning (62.19 ± 25.36), medication effect (65.21 ± 19.85), and overall QoL (62.04 ± 18.01) domains were fair. Participants had poor scores in the seizure worry (47.73 ± 24.62) and cognitive functioning (46.41 ± 25.32) domains (Table 3).

**Table 3:** QOLIE-31-P and subdomains QOLIE-31-P: patient-weighted Quality Of Life in Epilepsy-31; JME: juvenile myoclonic epilepsy The QOLIE-31-P score was poor in only 12 (27.91%) of the 43 patients. The mean value of the QOLIE-31-P score of study subjects was 62.29 ± 25.02, with a median (IQR) of 64.8 (45.95-79).

QOLIE-31-P and subscale domains	Mean score (± SD) of all JME patients (n = 43)	Good (%)	Fair (%)	Poor (%)
Seizure worry	47.73 ± 24.62	20.93	20.93	58.14
Overall well-being	63 ± 24.11	39.53	37.21	23.26
Energy/fatigue	65.56 ± 16.85	32.56	48.84	18.60
Cognitive functioning	46.41 ± 25.32	18.60	23.26	58.14
Medication effects	65.21 ± 19.85	32.56	44.19	23.26
Social functioning	62.19 ± 25.36	34.88	34.88	30.23
Overall QoL	62.04 ± 18.01	32.00	48.00	20.00
QOLIE-31-P score	62.29 ± 25.02	34.88	37.21	27.91

QOLIE-AD-48

The mean score (± SD) of the QOLIE-AD-48 score in adolescent study participants was fair (69.71 ± 13.13) and a majority (five, 71.4%) had fair QoL (Table 4). Scores of subdomains such as epilepsy impact (66.02 ± 13.07), memory (74.89 ± 4.5), and physical functioning (57.36 ± 18.94) were fair. The stigma perception (78.29 ± 17.86) score, social support (78.44 ± 16.14) and social behaviour (80.36 ± 18.67) subdomains had good scores, reflecting the lesser impact on QoL. The attitude toward epilepsy was fair (63.54 ± 14.68). The health perception of the QOLIE-AD-48 score of study subjects was fair (56.5 ± 16.9).

**Table 4:** QOLIE-AD-48 and subdomains in adolescent JME participants QOLIE-AD-48: Quality of Life in Epilepsy-Adolescents-48; JME: juvenile myoclonic epilepsy For five (71.43%) of the patients, the QOLIE-48-AD score was fair. The QOLIE-48-AD score was good in only two (4%) out of seven patients. The mean value of the QOLIE-48-AD score of study subjects was fair (69.71 ± 13.13), with a median (IQR) of 66.6 (60.15–77.05).

QOLIE-AD-48 and subscale domains	Mean score (± SD) of all JME patients (n = 7)	Good (%)	Fair (%)	Poor (%)
Epilepsy impact	66.02 ± 13.07	28.57	57.14	14.29
Memory	74.89 ± 4.5	57.14	42.86	0.0
Physical functioning	57.36 ± 18.94	28.57	42.86	28.57
Stigma	78.29 ± 17.86	85.71	0.0	14.29
Social support	78.44 ± 16.14	85.71	0.0	14.29
Social behavior	80.36 ± 18.67	71.43	28.57	0.0
Attitude	63.54 ± 14.68	28.57	57.14	14.29
Health perception	56.5 ± 16.9	14.29	42.86	42.86
QOLIE-48-AD score	69.71 ± 13.13	28.57	71.43	0.0

**Figure 1:**
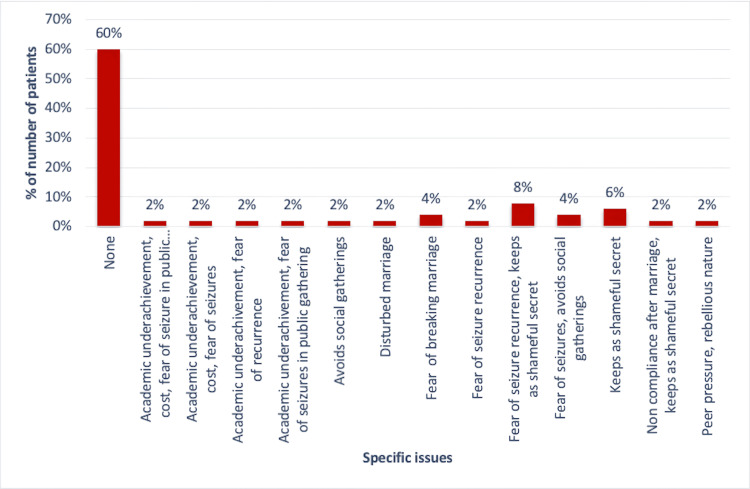
Distribution of psychosocial and emotional issues of JME study participants JME: juvenile myoclonic epilepsy

Four of the patients (8%) reported fear of seizure recurrence, and three (6%) perceived epilepsy as a shameful secret. Academic underachievement due to epilepsy was seen in one (2%) patient and one (2%) avoided public gatherings. In married JME participants, one (2%) reported a disturbed marriage, two (4%) expressed a fear of breaking their marriages, and one (2%) reported drug noncompliance after marriage (Figure 1).

Through screening using M.I.N.I. 7.0.2, a structured interview for DSM-5 and ICD-10, 14 (28%) screened positive for anxiety and seven (14%) screened positive for depression. They were further evaluated by DSM-5 for fulfilment of criteria for current and lifetime diagnosis. Psychiatric comorbidity was present according to DSM-5 classification criteria in only eight (16%) out of 50 patients (Table 5). Out of eight patients with psychiatric diagnoses, five (10%) of the patients had anxiety and three (6%) had depression.

**Table 5:** Comparison of clinical variables in JME participants with and without psychiatric comorbidity ^$^: Fisher’s exact test; JME: juvenile myoclonic epilepsy; GTCS: generalized tonic-clonic seizure No association was found between the age of onset of JME, gender, mono/polytherapy, and epilepsy control with the occurrence of psychiatric comorbidities (p-value > 0.05).

Variables	Psychiatric comorbidity (n = 8)	Without psychiatric comorbidity (n = 42)	P-value
Age of onset of seizures (years)			1^$^
11-20	8 (100.00%)	40 (95.24%)	
>20	0 (0%)	2 (4.76%)	
Gender			0.436^$^
Male	4 (50.00%)	14 (33.33%)	
Female	4 (50.00%)	28 (66.67%)	
Duration of seizures (years)			0.18^$^
≤5 years	4 (50.00%)	11 (26.20%)	
6-10 years	1 (12.50%)	19 (45.24%)	
11-15 years	1 (12.50%)	9 (21.42%)	
>15 years	2 (25.00%)	3(7.14%)	
Seizure characteristics and frequency			
GTCS (per year)			0.006^$^
<1	1 (12.50%)	25 (59.52%)	
1-3	4 (50.00%)	15 (35.71%)	
>3	3 (37.50%)	2 (4.76%)	
Myoclonic jerks (per month)			
Nil	1 (12.50%)	2 (4.76%)	0.31^$^
1-3	6 (75.00%)	37 (88.10%)	
>3	1 (12.50%)	3 (7.14%)	
Epilepsy control			1^$^
Yes	3 (37.50%)	17 (40.48%)	
No	5 (62.50%)	25 (59.52%)	
Drug therapy			0.056^$^
Monotherapy	2 (25.00%)	27 (64.29%)	
Polytherapy	6 (75.00%)	15 (35.71%)	

**Figure 2:**
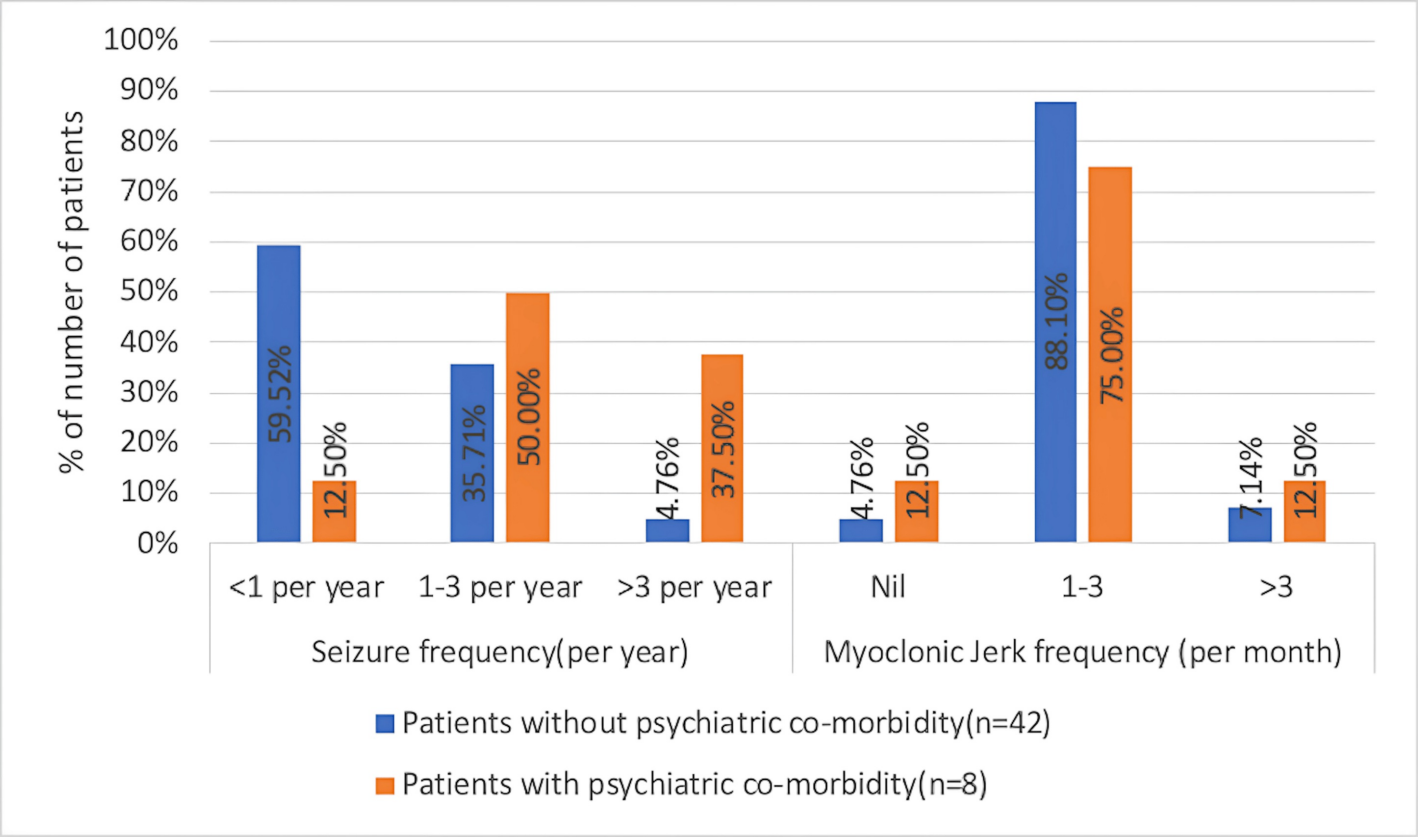
Association of seizure frequency and characteristics with occurrence of psychiatric comorbidities

No significant association was seen between sociodemographic variables and QoL (p-value > 0.05). No significant association was found between age of onset, duration of epilepsy, epilepsy control, or type of drug therapy with QoL (p > 0.05). There was no significant association found between the seizure frequency and perceived QoL (p = 0.598). The mean age of onset (in years) of anxiety was 15 ± 3 years, and for depression, it was 15 ± 2 years. Duration of epilepsy was less than or equal to five years in four (80%) of the participants with anxiety; one (20%) participant with anxiety and one (33.33%) participant with depression had a duration of epilepsy over 15 years. There was no association between the duration of epilepsy (years) and with occurrence of anxiety/depression (p = 0.071). GTCS frequency was 1-3 per year in four (50%) of participants and > 3 per year in three (37.50%) of participants in those with psychiatric comorbidity, which was significantly higher as compared to patients without psychiatric comorbidity (35.71% and 4.76%, respectively; Figure 2; Table 5). Significant association was seen in the distribution of GTCS seizure frequency (per year) with psychiatric comorbidity (p = 0.006). No significant association was seen in the distribution of myoclonic jerks frequency (per month) with psychiatric comorbidity (p = 0.31). Myoclonic jerks frequency (per month) was 1-3 in a majority of 37 (88.1%) patients without psychiatric comorbidity and six (75%) patients with psychiatric comorbidity, followed by > 3 in three (7.14%) of patients without psychiatric comorbidity and in one (12.50%) of patients with psychiatric comorbidity.

**Figure 3:**
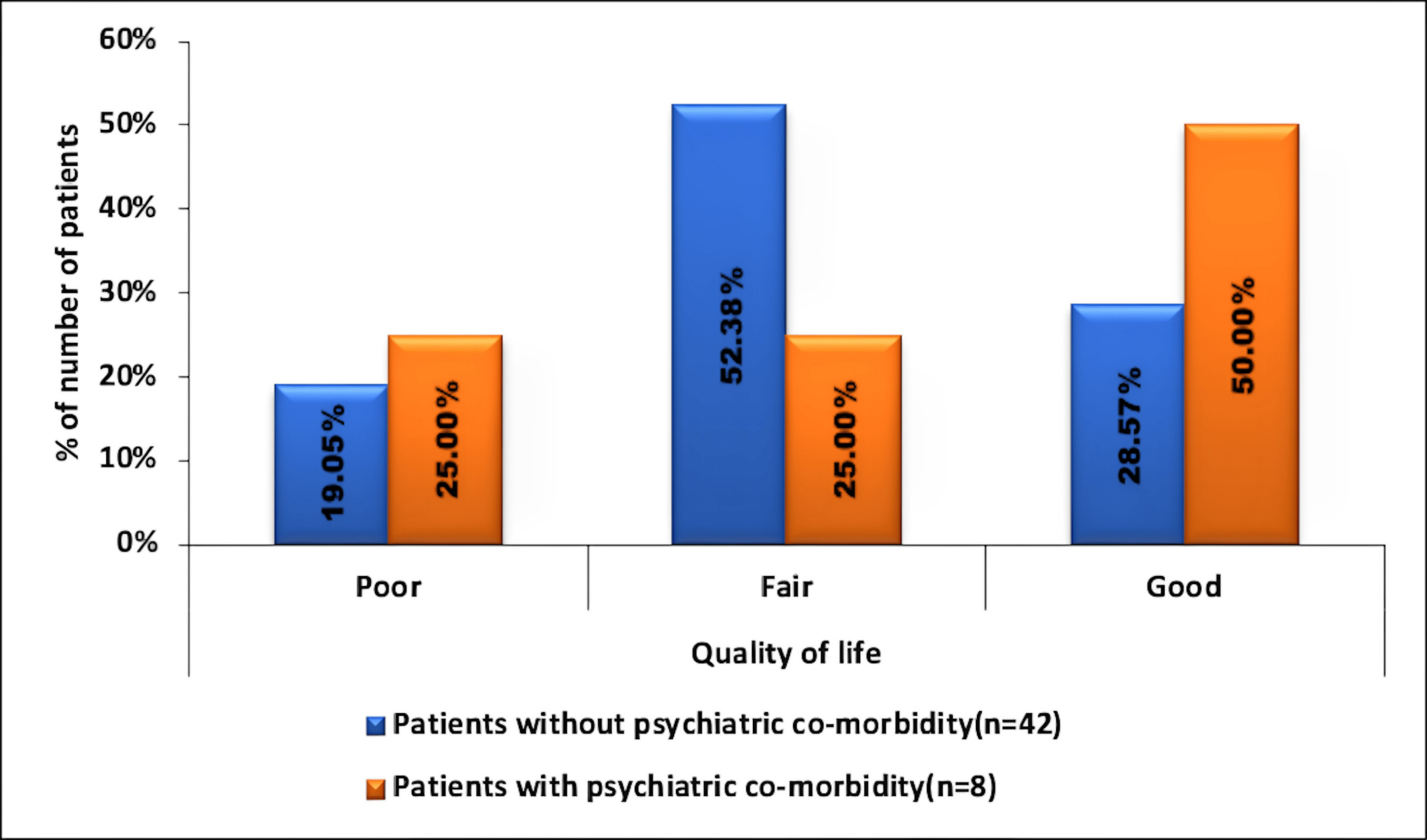
Association of QoL in JME with occurrence of psychiatric comorbidity No significant association was seen in the distribution of QoL with psychiatric comorbidity (p = 0.58, t-test). QoL was fair in 52.38% of participants without psychiatric comorbidity and in 25% with psychiatric comorbidity and good in 28.57% without psychiatric comorbidity and 50% with psychiatric comorbidity. QoL was poor in 19.05% of participants without psychiatric comorbidity and 25% of participants with psychiatric comorbidity, with no significant difference in distribution between them (p = 0.368; Fisher’s exact test). JME: juvenile myoclonic epilepsy; QoL: quality of life

The mean QOL in patients without psychiatric comorbidity was fair (61.42 ± 18.01). In those with psychiatric comorbidity, the mean QoL was also fair (65.31 ± 18.87). There was no significant association between QoL and the occurrence of psychiatric comorbidities in the JME participants (p = 0.58), as seen in Figure 3.

## Discussion

JME is a well-characterized subsyndrome of GGE that usually manifests during adolescence and is characterized by uni- or bibrachial myoclonic jerks, GTCS, and even absence seizures and is sometimes misdiagnosed as epilepsy with a focal onset [3-6]. Patients must be on treatment for prolonged periods, maybe even lifelong. Its occurrence in the prime years of life has a great impact on the QoL of these patients but has remained under-evaluated because of its categorization as an epilepsy syndrome with a good prognosis [11].

In our study, 96% of patients had an age of onset of habitual seizures between 11 and 20 years of age with a mean age of onset at 15.28 ± 2.9 years, comparable to the findings of previous authors [11-13]. Adolescents less than 17 years old with epilepsy are a special category because, in this transitional period, they form a sense of identity and peer relationships and make educational decisions. A majority of the participants in the study, 42% were students and had completed high school education but 8% were school dropouts due to reported fear of seizure recurrence in public places or due to academic underachievement. This is corroborated by the works of Schneider-von et al. and Dourado et al. [14,15]. School dropouts due to epilepsy-related factors continue to be an important problem and require awareness programs at schools for teachers, students, and parents. Every clinic visit requires counselling and education regarding epilepsy and drug compliance for both the patient and their caregiver.

On further analysis, we found two important determinants negatively affecting QoL in adolescent patients - namely, physical functioning (mean score = 57.36 ± 18.94) and health perception (mean score = 57.36 ± 18.94). This is comparable to the findings of Nagarathnam et al., Siqueira et al., and Stevanovic [16-18]. Psychological disturbances such as reduced energy and feeling inferior to others should be a priority for intervention to improve long-term outcomes in adolescents.

Further, the QOLIE-31-P score (mean score = 62.29 ± 25.02) was also fair in 37.21% of the adult patients. This is in accordance with the works of various authors such as Somayajula et al. (mean score = 68.70 ± 11.23) and Devinsky et al. (mean score = 67.2 ± 18.4) and much better than the findings of Guekht et al. (mean score = 42.1 ± 3.8) [19-21]. Even though JME is a lifelong condition, repetitive visits and a long and healthy doctor-patient relationship might have had a protective effect on the overall QoL of these patients. We also found that the seizure worry domain was poor in 58% of the adult JME patients (mean = 47.73 ± 24.62). This domain is much more affected as compared to the works of authors such as Holtkamp et al. (seizure worry subscale score = 89 ± 2.8) and Somayajula et al. (seizure worry subscale score = 79.8 ± 19.3) [5,19]. This can be explained by the psychological response and emotional trauma of seizures, which have the greatest impact on perceived QoL. The cognitive functioning domain was also poor (46.41 ± 25.32) in 58.14% of the adults with JME. JME is associated with thalamo-frontal circuit disturbances, which might affect cognitive functioning as shown in recent literature [3,6].

Epilepsy is known to be associated with lower employment rates and stigmatization in the workplace [1,2]. We could not establish any significant correlation between employment status and QoL (p=0.729). Our findings were similar to the works of Holtkamp et al. and Somayajula et al. [5,19]. Studies have shown that patients with JME have difficulties in maintaining long-term relationships and have disturbed marital lives [6,13,22]. In our study, 30% of the JME participants were married, 4% expressed fear of a breaking of their marriages due to epilepsy, 2% reported disturbed marriages, and 2% reported drug noncompliance after marriage. Our findings echo those of Kim and Shetty et al. [22,23]. We suggest that building a strong social network and establishing spousal support are necessary for a favourable outcome in PWE.

Seizure frequency has been stated previously to be the strongest predictor of QoL [14,18,24]. An occurrence of more than 10 GTCSs per year reduces the chances of seizure freedom and increases the chances of social isolation in the long term. We found that 20% of patients with a GTCS frequency of over three years had a poor QoL and 37.5% of those with a GTCS frequency of 1-3/year had a fair QoL. No significant association was found between seizure frequency and QoL (p =0.598). Our finding differs from the previous works of Schneider-von et al., Moschetta and Valente, and Senol et al. [14,25,26]. We can say that QoL is a self-perception and, apart from adequate seizure control, patients of JME should be counselled by healthcare providers so that they can live their lives as productively as people without epilepsy.

We also found that higher GTCS seizure frequency is an independent risk factor for the development of psychiatric comorbidities in JME (p =0.006). Similar findings were shown by Filho et al. and Ertem et al. [27,28]. This is explained by the fact that psychiatric disorders share common neural pathways with epilepsy, facilitating the occurrence of one in the presence of the other. Abnormal functioning of the hypothalamic-pituitary axis, decreased secretion of serotonin in the central nervous system and decreased binding of serotonin in the anterior and posterior cingulate and raphe magnus are some of the described mechanisms.

JME is a treatable but lifelong epilepsy syndrome. This uniqueness has been attributed to hamper QoL in these patients. In this study, most patients had a disease duration of 9.04 ± 7.14 years, with 45.83% having a fair QoL. This is in concordance with the works of Holtkamp et al. and Shetty et al. [5,23]. Many studies have shown that the longer the duration of epilepsy, the more the occurrence of psychiatric comorbidities. In this study, however, most participants (50%) with psychiatric comorbidities had a duration of epilepsy less than five years without any statistically significant correlation between the duration of epilepsy and psychiatric comorbidities (p = 0.18). Our findings were similar to the works of Ertem et al. and Trinka et al. [28,29]. We can conclude that epilepsy and psychiatric comorbidities should be treated as a spectrum disorder rather than separate entities and comprehensive epilepsy care should also include liaison psychiatry for screening and treatment of psychiatric comorbidities. This study reiterates the need for cognitive behavioural therapy and psychotherapy in those with psychiatric comorbidities.

On analysis of the psychiatric comorbidities of our study population, the prevalence of psychiatric disturbances was 16%. Of them, 10% were classified as anxiety and 6% as depression according to the DSM-5 criteria of diagnosis. The findings were similar to the works of Holtkamp et al. (24.4%), Somyajula et al. (46.6%), and Filho et al. (49%) [5,19,27]. This may be due to the small sample size of our study and the variability of methods by which psychiatric diagnoses were assigned in previous studies, such as the Structured International Classification of Diseases (SICD) and DSM criteria-based interviews; unstructured, subjective questionnaires; and scale-based surveys, which tend to overrate the problems.

There is a traditional view that depressive disorders in epilepsy are more common over 30 years of age [30]. However, in this study, we found that psychiatric comorbidities were more prevalent in the age groups of less than 20 years and over 30 years. Anxiety and depression were equally distributed across both of these age groups. This is in accordance with the findings of Somayajula et al. and Trinka et al. [19,29]. We concluded that age is not a determinant for the occurrence of psychiatric comorbidities in JME (p = 0.072). We may further say that frontal lobe dysfunction intrinsic to JME may contribute to comorbid psychiatric disorders.

The strength of the study was the implementation of a standardized QoL inventory. In the previous studies, the association between epilepsy and psychiatric comorbidities was examined frequently, but studies that evaluated QoL parameters - including functioning, physical health, psychosocial health, degree of being independent, and social relationships - are sparse. We have assessed the QoL in adolescents and adult JME participants separately which is a major strength. Thus, our study can act as a stepping zone for further larger studies. The question of whether the high prevalence of psychiatric comorbidities is specific to JME or is also present in other focal or generalized epilepsies, cannot be answered by this study because this study did not include a control group of other types of epilepsies. The psychiatric assessment in our study was a one-time assessment. Even though the M.I.N.I 7.0.2 screened for anti-social personality disorder, it did not screen for other personality types defined in DSM-5 and this remains a possible limitation of the study. Also, our study was a single-centre study. A multicentric study comprising many patients should be conducted to gain more information about QoL and psychiatric comorbidities in patients with JME.

## Conclusions

JME has a negative impact on the overall QoL in both adults and adolescents. There are different determinants of overall QoL in adults and adolescents with JME. Physical functioning and overall health perception affected the QoL in adolescent JME participants. Fear of seizure recurrence and cognitive functioning affected the QoL in adult JME. We found that socio-demographic factors, age of onset, duration of epilepsy, seizure characteristics and frequency, control of epilepsy, anti-seizure drug compliance, and monotherapy/polytherapy did not impact the overall QoL. Cultural and age-specific programs should be undertaken to address these issues in different age groups to reduce the stigma associated with epilepsy and ensure better compliance with anti-seizure medications. Anxiety and depression were the comorbid psychiatric disorders. Seizure frequency was an independent risk factor for the development of psychiatric disorders. The presence of these comorbid conditions must be addressed and treated holistically as a part of comprehensive epilepsy care to improve the long-term prognosis. In view of this, population‐based longitudinal studies focusing on the psychosocial and psychobiological aspects and psychiatric comorbidities in JME are warranted.
